# Investigation of Antidepressant Properties of Yohimbine by Employing Structure-Based Computational Assessments

**DOI:** 10.3390/cimb43030127

**Published:** 2021-10-27

**Authors:** Munazzah Tasleem, Abdulwahed Alrehaily, Tahani M. Almeleebia, Mohammad Y. Alshahrani, Irfan Ahmad, Mohammed Asiri, Nadiyah M. Alabdallah, Mohd Saeed

**Affiliations:** 1 School of Electronic Science and Engineering, University of Electronic Science and Technology of China, Chengdu 610054, China; 2 Department of Biology, Faculty of Science, Islamic University of Madinah, P.O. Box 170, Madinah 42351, Saudi Arabia; abdulwahed.alrehaily@iu.edu.sa; 3 Department of Clinical Pharmacy, College of Pharmacy, King Khalid University, P.O. Box 61413, Abha 62529, Saudi Arabia; talmelby@kku.edu.sa; 4 Department of Clinical Laboratory Sciences, College of Applied Medical Sciences, King Khalid University, P.O. Box 61413, Abha 62529, Saudi Arabia; moyahya@kku.edu.sa (M.Y.A.); irfancsmmu@gmail.com (I.A.); masiri@kku.edu.sa (M.A.); 5 Department of Biology, College of Science, Imam Abdulrahman Bin Faisal University, P.O. Box 1982, Dammam 31441, Saudi Arabia; nmalabdallah@iau.edu.sa; 6 Department of Biology, College of Sciences University of Hail, P.O. Box 2440, Hail 2440, Saudi Arabia

**Keywords:** yohimbine, molecular docking, site directed mutational studies, MD simulation, ADMET

## Abstract

The use of pharmaceuticals to treat Major Depressive Disorder (MDD) has several drawbacks, including severe side effects. Natural compounds with great efficacy and few side effects are in high demand due to the global rise in MDD and ineffective treatment. Yohimbine, a natural compound, has been used to treat various ailments, including neurological conditions, since ancient times. Serotonergic neurotransmission plays a crucial role in the pathogenesis of depression; thus, serotonergic receptor agonist/antagonistic drugs are promising anti-depressants. Yohimbine was investigated in this study to determine its antidepressant activity using molecular docking and pharmacokinetic analyses. Additionally, the in silico mutational study was carried out to understand the increase in therapeutic efficiency using site-directed mutagenesis. Conformational changes and fluctuations occurring during wild type and mutant serotonergic receptor, 5-hydroxytryptamine receptors 1A (5HT1A) and yohimbine were assessed by molecular dynamics MD simulation studies. Yohimbine was found to satisfy all the parameters for drug-likeness and pharmacokinetics analysis. It was found to possess a good dock score and hydrogen-bond interactions with wild type 5HT1A structure. Our findings elaborate the substantial efficacy of yohimbine against MDD; however, further bench work studies may be carried out to prove the same.

## 1. Introduction

Major Depressive Disorder (MDD) is a commonly occurring serious mood disorder affecting more than 264 million people that plagues socioeconomic burden across the world. In 2008, the World Health Organization (WHO) identified major depressive disorder (MDD) as the third leading cause of disease burden worldwide [1,2,3]. Females suffer from depression at a higher rate (5.1%) than males (3.6%). MDD becomes a serious health condition when it runs for a long time with moderate to severe intensity [1]. In India, MDD is one of the leading causes of non-fatal disease burden, where 197.3 million people are affected by mental disorders, including 45.7 million people with depressive disorder and 44.9 million people with anxiety disorder [4]. These disorders not only affect the patient but also affect their caretakers, stagnating their productivity at work and damaging their social relationships, thus increasing the financial burden on society [2,5]. According to the Diagnostic and Statistical Manual of Mental Disorders-V edition (DSM-V) [6], there are over 450 psychiatric disorders currently endured by people around the globe. MDD or clinical depression is one of those psychiatric disorders which are least studied and was observed as the third leading cause of disability in 2015 [7]. MDD is a significant challenge for neurobiology and psychiatry for its overlapping symptoms and diverse etiologies, along with an inadequate understanding of neurobiological basis [8,9]. The “Monoamine Theory”, is one of the most well-known and commonly accepted theories, which states that three neurotransmitters, serotonin, dopamine, and norepinephrine, are responsible for depressive symptoms in people [10]. According to the theory, down regulation of the three neurotransmitters in the synaptic cleft of the brain leads to feeling low, irritated, agitated, sad, and other symptoms of depression. Although the primary cause of depression is unknown, it is frequently stated that a person’s depression might be caused by biological, social, environmental, or psychological factors, and that no single gene variant is responsible for its pathogenesis [11]. Overall, the underlying pathology of depression is heterogeneous, due to which a complete fixed treatment of depression is not yet available [9].

Various approaches, including monoamine oxidase inhibitor (MAOI), tricyclic anti-depressant (TCA), NK-1antagonist, GR antagonists, melatonergic drugs, and somatic therapy, were used to treat depression [12]. Other therapies for the treatment of depression are psychotherapy viz. the talk therapy [13,14], electroconvulsive therapy (ECT) [15], neuronetics NeuroStar Transcranial Magnetic Stimulation (TMS) System, a class II medical device for producing magnetic field [16], and Yoga, which has proved to be effective for coping with mental diseases [17]. Due to a lack of awareness of depression, diagnosis and therapy are delayed, reducing the therapeutic benefit of medications. Moreover, the available allopathic medicines result in side effects that range from nausea, sedation, somnolence, changes in appetite, problems in sleeping, vision problems, skin dryness, diarrhea, sexual problems, bladder problems, metabolic dysregulation, weight gain, akathisia, cardiac problems, shivering, dyslipidemia, hypertension, hyperglycemia, and low blood pressure to the occurrence of suicidal thoughts [18,19]. Therefore, there is an immediate requirement for an alternative pharmacotherapy with high efficacy and low toxicity. Since ancient times, herbal medicines have been used to treat psychological diseases due to their high efficacy and minimal or no side effects compared to their synthetic counterparts [20,21].

Yohimbine, a small indole alkaloid, present in the bark of Central African tree- *Pausinystalia yohimbe* [22], is used to treat various diseases, including marijuana abuse, male erectile dysfunction, diabetes mellitus type II, idiopathic orthostatic hypotension, and depression [23,24,25,26,27,28,29]. Yohimbine is an alpha-2-adrenoceptor antagonist possessing activity in the central and peripheral nervous system [22]. To use yohimbine in anti-depressive pharmacotherapy, its pharmacokinetic and pharmacodynamic properties are to be well investigated [30].

**Figure 1:**
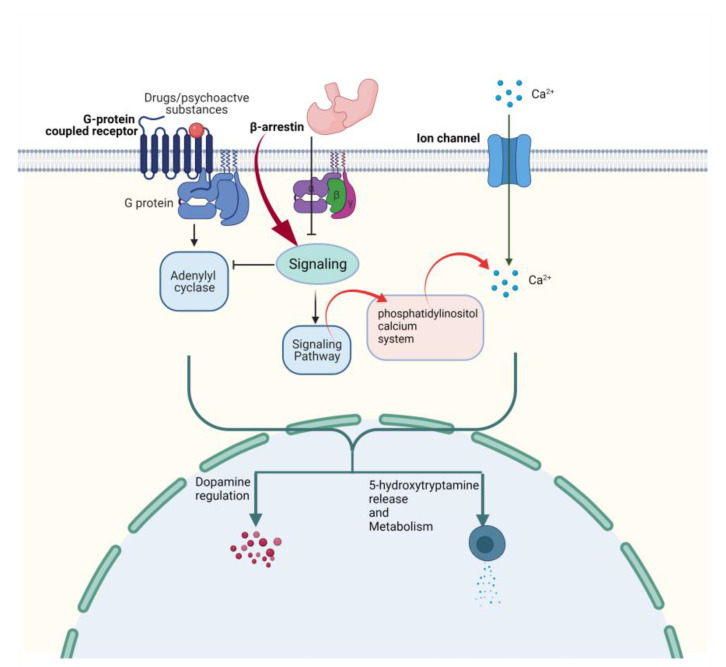
5HT1A regulation affected by G-protein coupled receptors, β-arrestin, and Ca^+^.

In the human genome, G protein-coupled receptors (GPCRs) belong to the largest family of membrane proteins and are the target of maximum therapeutic drugs and have been documented to be involved in the progression of depression [31]. Membrane proteins (MPs) encode approximately 30% of the human genome, play a key role in performing biological functions, and target most of the available drugs. GPCRs involved in depression are noradrenergic receptors [20,21,22,23], serotonergic receptors, dopaminergic receptors [24], protein group [25], glutamate receptors (mGlus), and gamma-aminobutyric acid type B (GABA(B)) [32,33]. The 5-hydroxytryptamine G-protein-coupled receptor also serves as a receptor for various drugs and psychoactive substances. Ligand binding activates signaling through guanine nucleotide-binding proteins (G proteins) and regulates downstream effectors like adenylate cyclase. Members of the beta-arrestin family block G protein-mediated signaling while activating alternative signaling pathways. It plays a key role in the metabolism and release of 5-hydroxytryptamine and regulates dopamine, thereby regulates neuronal activity, mood, and behavior, as shown in Figure 1 [34]. Millen et al. studied yohimbine against α_2_ ARs(GPCRs) and observed it to be partial agonist h5-HT(1A), partial agonist/antagonist at h5-HT(1B), and h5-HT(1D), and as an antagonist against hD(2) receptor [35]. The majority of selective 5-HT1A agonists that have been developed to date have failed to prove clinical efficacy. Despite claims the contrary, the therapeutic effectiveness, and use of the only 5-HT1A agonist currently on the market (e.g., buspirone) lag far behind other antidepressants. Presynaptic 5-HT1A autoreceptors, on the other hand, are a primary target of antidepressants that either elevate extracellular 5-HT (SSRIs, MAOIs) or take actions on such receptors. Postsynaptic 5-HT1A receptor activation in corticolimbic areas shows promise for the therapeutic efficacy of antidepressant medications. Therefore, 5-HT1A receptors play an integral role in the pathogenesis and treatments of psychiatric disorders, including the pre-and postsynaptic elements of serotonergic transmission. However, with a few notable exceptions, molecules targeting 5-HT1A receptors have performed poorly to live up to the high expectations placed on them in treating these disorders. New antidepressant medications should target the postsynaptic receptors or intracellular signaling pathways to maximize the therapeutic effects of existing antidepressant medications. As a result, they would circumvent adaptive neuronal mechanisms (pre-and postsynaptic) that impede their therapeutic effects. The discovery and development of new 5-HT1A receptor agonists selective for postsynaptic 5-HT1A receptors could open up new avenues in the field [36,37]. In this study, we explored anti-depressive properties of yohimbine to analyze the binding potential with specific serotonergic targets by applying state of the art in silico approaches viz., homology modeling, molecular docking, MD simulation, and interaction studies. We used in silico site-directed mutagenesis to assess the increase in the stability centers of the target protein, which can have a large impact on yohimbine and potential antidepressants binding.

## 2. Materials and Methods

### 2.1. ADMET Profile of Yohimbine

Yohimbine (C_21_H_26_N_2_O_3_) 3D structure was retrieved from PubChem with ID- 8969 (https://pubchem.ncbi.nlm.nih.gov/ (accessed on 2 February 2021)) [38]. Pharmacokinetic properties of yohimbine were calculated using the ‘ADMET descriptor’ protocol from Discovery Studio (DS) programme (Dassault Systemes, v 20.1, BIOVIA Corp., San Diego, CA, USA) (https://www.3ds.com/products-services/biovia/products/molecular-modeling-simulation/biovia-discovery-studio/ (accessed on 2 February 2021)) [39,40] and an online tool ‘ADMETlab’ (https://admet.scbdd.com (accessed on 2 February 2021)) supported by CBDD group [41]. Toxicity and drug-likeness were estimated by Toxicity prediction ‘TOPKAT’ module and ‘Filter by Lipinski’s and Veber’s Rule’ module from DS [42].

### 2.2. Protein Selection of MDD and Sequence Analysis

Target proteins (TPs) for MDD that belongs to GPCRs include alpha(2)-AR, serotonin receptors (5HT1A, 5HT1B, 5HT1D, 5H2A, 5H2B, 5H2C), dopamine receptors (D2 and D3) and ATP sensitive protein group [35]. The experimentally determined structures of 5HT1A are 7E2X, 7E2Y, and 7E2Z; however, these structures are solved with electron microscopy at a low resolution of 3 Å and more, which show only the basic contours of the protein chain. Additionally, these three-dimensional structures lack continuity in the structure and are broken at two stretches of the amino acid sequence, viz. stretch1: between Trp175 and Pro184, and stretch2: between Lys228 and Lys324. In the present study, 5HT1A was selected for in silico studies to understand the role of important active site residues interacting with yohimbine. To generate the missing residues, structure protein sequence of 5HT1A was retrieved from the Universal Protein Resource (UniProt) (https://uniprot.org/ (accessed on 1 February 2021)) [43]. A pair-wise sequence similarity search with sequences of known 3D structure using NCBI-Protein Blast (https://blast.ncbi.nlm.nih.gov/ (accessed on 1 February 2021)) with PDB database was carried out to identify the reference template sequence [44].

#### 2.2.1. Physicochemical Characterizations and Function Prediction

Physico-chemical parameters of 5HT1A were calculated through Expasy’s ProtParam server (https://web.expasy.org/protparam/ (accessed on 1 February 2021)) [45]. To assign precise functions to the TP, Conserved Domain Database (CDD) (https://www.ncbi.nlm.nih.gov/cdd/ (accessed on 1 February 2021)) [46], SMART (Simple Modular Architecture Research Tool) (http://smart.embl-heidelberg.de/ (accessed on 1 February 2021)), SIFTER (https://sifter.berkeley.edu/ (accessed on 1 February 2021)), ScanProsite (https://prosite.expasy.org/scanprosite/ (accessed on 1 February 2021)), and CATH were employed. SMART (Simple Modular Architecture Research Tool) identifies compositionally biased regions such as trans-membrane, coiled-coil, and signal peptide through multiple sequence alignment [47,48,49,50]. SIFTER (Statistical Inference of Function Through Evolutionary Relationships) is a gene molecular function prediction algorithm annotating throughout the phylogenetic tree based on a statistical model of function evolution [51]. ScanProsite identifies PROSITE signature matches in protein sequences to find functional and structural intra-domain residues, improving the power of function prediction [52]. The CATH database provides structurally related proteins despite low sequence identity to produce a hierarchical classification of protein domains based on their folding patterns [53]. Protein functions are modulated by post-translational modifications (PTMs), which were detected through iPTMnet (https://research.bioinformatics.udel.edu/iptmnet/ (accessed on 1 March 2021))—an integrative bioinformatics approach to produce rich PTM information, such as PTM enzyme–substrate-site relationships, PTM-specific protein–protein interactions (PPIs), and PTM conservation across species [54].

#### 2.2.2. 3D Structure Generation and Refinement

The identified reference template was selected for generating three-dimensional structure of the TP through homology modeling approach [55]. Psi-Pred software (http://bioinf.cs.ucl.ac.uk/psipred/ (accessed on 1 April 2021)) consists of three programs—PSIPRED: to predict secondary structure; GenThreader: to predict protein fold; MEMSAT 2: to predict topology and structure of the protein, and was used to predict secondary structures and topology of the TP sequences (http://www.ebisu.co.uk/chemogenomix.com/chemogenomix/GenThreader.html (accessed on 1 April 2021)) [56]. Homology modeling tools such as MODELLER version 9.2 (https://salilab.org/modeller/ (accessed on 28 September 2021)), Swiss-Model (https://swissmodel.expasy.org/ (accessed on 1 April 2021)), pGenTHREADER from PsiPred server, and Phyre 2 (http://www.sbg.bio.ic.ac.uk/phyre2/ (accessed on 28 September 2021)) were used to generate the modelled structure of the TP for functional characterization [57,58,59,60]. Since no single quality measure is perfect, the generated models were validated by ProQ (https://proq.bioinfo.se/ProQ/ (accessed on 1 April 2021)), ProSA (prosa.services.came.sbg.ac.at/), ERRAT (https://servicesn.mbi.ucla.edu/ERRAT/ (accessed on 28 September 2021)), and PROCHECK (https://www.ebi.ac.uk/thornton-srv/software/PROCHECK/ (accessed on 1 April 2021)). ProQ is a neural-network-based approach to predict the local and global quality of protein models, such as frequency of atom–atom contacts, and measure LG or MaxSub score to assess the model quality [61]. ProSA (Protein Structure Analysis) tool, on the other hand, identifies potential errors in the structure based on statistical analysis on all available protein structures [62]. ERRAT tool is based on FORTRAN that successfully identifies the random distribution of atoms in protein structure to produce incorrect regions [63]. Validation of geometry of Cα and deviation of the observed Cβ atom from the ideal position to explore the bond angle distortion in the modeled structures was evaluated by PROCHECK server [64,65].

### 2.3. Identification of Binding Pocket and Protein-Molecular Docking Studies

The binding pocket of the TP model predicted by the module ‘PDB Site Records’ in the “Define Site” protocol from DS was selected for docking procedure using the GOLD suite (https://www.ccdc.cam.ac.uk/solutions/csd-discovery/Components/Gold/ (accessed on 1 May 2021)) [66]. TP structure was prepared, and a cavity of 10 Å radius with XYZ coordinates of −25.479000, −5.895000, and 45.678000, respectively, was set around the predicted binding pocket. For predicting the best ligand binding pose, Gold Fitness Score was selected to measure and rank the ligand conformations. yohimbine was docked in the binding pocket using the GOLD Suite. Intra-molecular interactions formed within the docked complex were analyzed with ‘View Interactions’ tool from DS.

### 2.4. Comparative Analysis of 5HT1A Structure

The best homology modeled structure of 5HT1A was compared with available three-dimensional structure at RCSB protein data bank (https://www.rcsb.org/ (accessed on 20 September 2021)) and AlphaFold structure prediction (https://alphafold.ebi.ac.uk/ (accessed on 20 September 2021)) [34].

### 2.5. In Silico Mutant Preparation

Identified active site residues forming hydrogen bonds with yohimbine were selected for exploring the effect of a mutation in stability of docked complex and affinity of the binding ligand. Hydrogen bond-forming residues were mutated to the residues contributing to the stability of the protein structure by ‘Build Mutant’ protocol of the DS. The ‘Build Mutant’ tool is based on Modeller programme that mutates residues to the specified type; also, it optimizes the overall conformation of the mutated and neighboring residues. Further, conformation and orientation of the residues were corrected by refining side chains of the residues [57]. Mutant modeled structure was docked with yohimbine to study the role of predicted active site residues by analyzing intra-molecular interactions. The generated mutant modeled structure of 5HT1A was subjected to energy minimization with default parameters by applying CHARMm forcefield42 from ‘smart minimizer’ algorithm in the DS programme. Following energy minimization, flexible molecular docking was performed using GOLD Suite, and a cavity around the identified binding site using ‘Define Site’ protocol from DS was generated by adjusting radius of 10 Å in X, Y, and Z directions. The Gold Fitness Score was selected to estimate and rank the conformations of ligand to obtain the best pose after accurate binding conformation. The ligand pose with the highest Gold Fitness Score was analyzed for intra-molecular interaction studies.

### 2.6. Molecular Dynamics Simulation Study

For a comprehensive study of the effects of mutations on the 5HT1A structure, all-atom MD simulations were carried out for 60 ns under explicit water solvent conditions. MD Simulation was carried out with the GROMACS 4.6.5 (http://www.gromacs.org/ (accessed on 3 March 2021)) simulation suite at 300 K at the molecular mechanics level using the CHARMM36 force field to understand the factors behind the efficiency of yohimbine in inhibiting 5HT1A [67,68,69]. All systems, including 5HT1A and the complex of wild type 5HT1A with yohimbine, and mutant 5HT1A with yohimbine, were submerged in a dodecahedron box a simple point charge (SPC) water model [70] filled with water molecules, with borders defined using the *gmx editconf* module and salvation provided by the *gmx solvate* module. Further, *gmx genion* module was applied to neutralize the system by incorporating Na^+^ (sodium) and Cl^−^ (chloride) and preserving a physiological concentration at 0.15 M. The systems were subjected to 5000 steps of steepest descent minimization to eliminate any steric conflicts. This process was repeated until the maximum force of less than 1000 kJ/mol/nm was reached. All of the systems were further equilibrated at a constant temperature of 300 K and a pressure of 1 bar by deploying NVT using the V-rescale thermostat and NPT using the Parrinello–Rahman barostat ensemble process for 100 ps at a constant temperature of 300 K and a pressure of 1 bar [71,72]. A total of three MD simulations were performed for (i) 5HT1A modeled structure, (ii) 5HT1A modeled structure docked with yohimbine (5HT1AComplex_W), and (iii) 5HT1A mutant structure (N404L) docked with yohimbine (5HT1AComplex_M). Final simulation for each system was performed for 60 ns using a leapfrog integrator to acquire the time evolution of trajectories [73]. The resulting trajectories were analyzed using *gmx rms*, *gmx rmsf*, *gmx sasa*, *gmx gyrate*, and *gms trjconv* modules of GROMACS. The outputs were plotted into graphs using X-GRACE (GRaphing Advanced Computation and Exploration of data), and the coordinates retrieved at specific intervals were analyzed in Discovery Studio Visualizer.

## 3. Results and Discussion

### 3.1. ADMET Estimations

**Figure 2:**
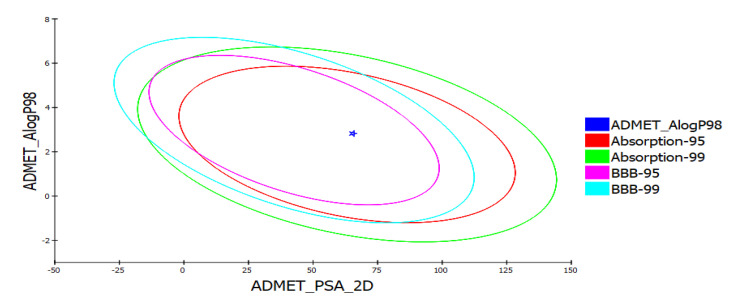
ADMET analysis of yohimbine showing plot of polar surface area (PSA) versus ALogP with 95% and 99% confidence limit ellipses referring to the blood–brain barrier (BBB) and intestinal absorption.

A compound absorbed through the gastrointestinal tract to be available in circulation, metabolized by metabolic enzymes to be excreted from the body, and does not interfere with normal biological processes, is considered to possess a good ADME profile [74]. Aqueous solubility predicted using a linear regression model in water at 25 °C was at level “2” indicating moderate solubility. Moderate distribution and permeability for yohimbine was assessed based on the predicted value of distribution coefficient D. Distribution coefficient P estimated for yohimbine revealed it to be an optimal lipid bilayer permeable. All the values associated with absorption, such as Papp (Caco-2 Permeability), Pgp-inhibitor (P-glycoprotein inhibitor), PgP-substrate, human intestinal absorption, and bioavailability indicated its good absorption. Parameters calculated to assess distribution properties of the yohimbine revealed it to distribute evenly and bind to tissues such as proteins and lipids and is highly lipophilic, as shown by good plasma protein binding (PPB), volume distribution (VD), and blood–brain barrier (BBB). A compound crossing the blood–brain barrier (BBB) is not considered safe for administration. BBB penetration level was predicted for yohimbine, indicating medium penetration. ADME model that uses 2D PSA and AlogP98 descriptors with 95% and 99% confidence ellipses were applied to predict intestinal absorption and blood–brain barrier penetration. The enclosed region within these ellipses defines the well-absorbed compounds region [42]. Drug metabolism depends upon CYP450 (cytochrome 450) enzymes and their isoforms. Since these enzymes are responsible for detoxification, molecules inhibiting these enzymes can cause toxicity [75]. In the liver, CYP2D6 accounts for only 2% of the total CYP content, however, it is responsible for the biotransformation of 20% of the drugs that undergo hepatic metabolism. The CYP2D6 inhibitors are lipophilic to a moderate degree. It was discovered that there are 57 different CYP isoforms in mammals, all of which are involved in the breakdown of certain molecules, including xenobiotics and endogenous compounds via oxidative metabolism. Clinical drug metabolism is carried out majorly by five CYP isoforms (3A4, 2D6, 2C19, 2C9, and 1A2) which are involved in the metabolism of more than 80 percent of the drugs used in clinical trials. Since yohimbine became an inhibitor of CYP2D6, it may impede the biotransformation of drugs metabolized by CYP450 enzyme. Calculation of other important ADME properties of yohimbine is listed in Table 1 and Table 2. Yohimbine falls inside the ellipses filter describing its good intestinal absorption and medium BBB penetration as shown in Figure 2. Yohimbine toxicity screening through TOPKAT and ADMETlab shows that predicted carcinogenicity values are within the expected range, and the compound possesses no risk of mutagenicity. However, it shows mild skin irritancy, severe ocular irritancy, and if consumed for long term or at high doses, it may impose developmental or reproductive toxicity in humans. Other toxicity screening parameters, including Rat inhalation LC_50_, Rate of Oral LD50, Rat chronic LOEAL, Daphnia EC50, and Fathead minnow LC_50_ are summarized in Table 3.

### 3.2. Sequence Analysis of Target Protein

**Figure 3:**
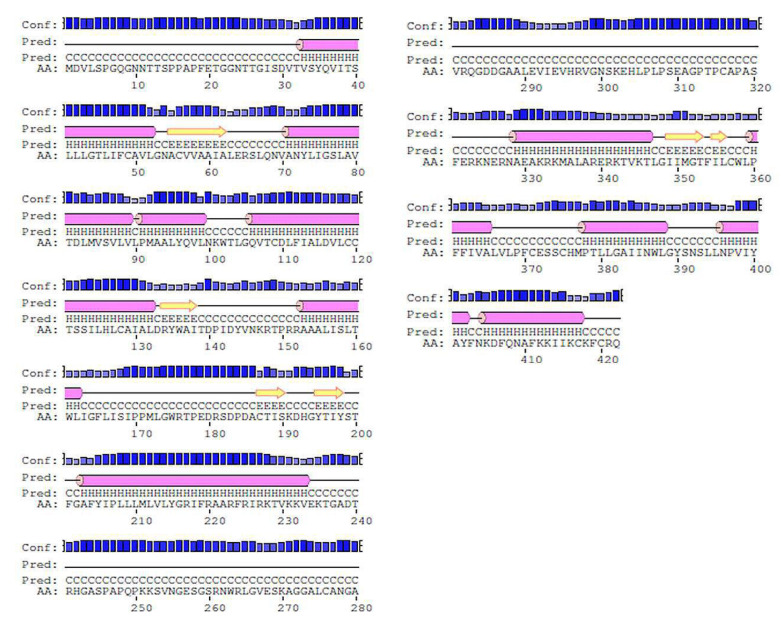
Secondary structures of 5HT1A retrieved through Psi-Pred shows presence of 42.65% helices as shown in pink cylinder, 52.13% sheets as shown in yellow arrow, and 6.87% loops as solid black connecting line.

An X-ray determined three-dimensional structure of high resolution with coordinates the of whole sequence is not yet available, FASTA sequence of 5HT1A was retrieved from UniProt (UniProt ID: P08908) which consists of 422 amino acid residues. The FASTA sequence was compared with sequences of known 3D structure with the highest similarity and query coverage using BlastP algorithm of NCBI BLAST in the PDB database. The top three structures with highest similarity to the target protein (TP) obtained were 7E2X_A, 7C61_A, and 4IAR_A. Since 7E2X is a low resolution, electron microscopic synthetic construct, and 7C61 is an X-ray structure of 3 Å resolution, therefore, these structures were excluded from the study. 5HT1A possess 41% identity and 91% query coverage with 4IAR_A [63]. Two agonist binding domains of three and five residues, ‘DRY’ and ‘NPxxY’ motif, were determined by using Conserved Domain Database (CDD) (which is the most widely used resource for protein annotation that includes manually curated domain models to describe protein sequence, structure, and function) [46], Simple Modular Architecture Research Tool (SMART) (which is a resource for protein identification and annotation that includes manually curated models for a large number of protein domains and is synchronized with protein databases, such as UniProt, Ensembl, and STRING) [47], Statistical Inference of Function Through Evolutionary Relationships (SIFTER) [51], ScanProsite [52], and Class Architecture Topology and Homologous superfamily (CATH) [53] in the TP. ‘DRY’ motif is three residues long whilst ‘NPxxY’ motif constitutes five residues. The presence of these domains and motifs in the TP discerns their role in efficient protein folding and functioning. Physico-chemical parameters such as atomic composition, molecular weight, isoelectric point, extinction coefficient, aliphatic index, instability index, and grand average of hydropathicity (GRAVY) of 5HT1A were computed by Expasy’s ProtParam Server [45]. An instability index of <40 was computed for the TP, indicating it to be a stable protein. Other predicted physico-chemical parameters are summarized in Table 4. iPTMnet tool [54] used to identify post-translational modifications (PTMs) in the TP explains the presence of phosphorylation, acetylation, and ubiquitination as presented in Table 5. Secondary structures and topology for 5HT1A obtained through Psi-Pred shows the presence of 42.65% helices, 52.13%, sheets, and 6.87% loops, as shown in Figure 3.

### 3.3. Structure Prediction and Quality Assessment

**Figure 4:**
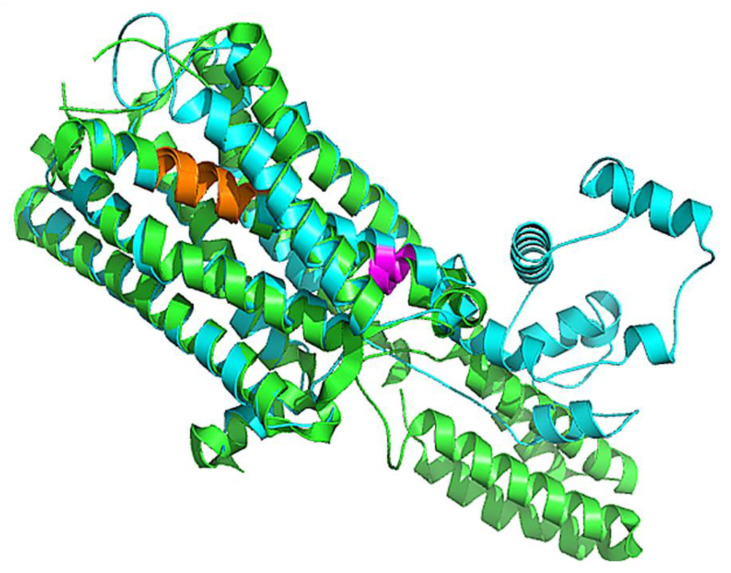
Superimposed structure of 5HT1A best model (cyan color) over the template structure of 4IAR_A (green color) in cartoon representation with 0.66 Å RMSD. Highly conserved agonist binding domains are shown in orange and magenta colors.

Various homology modeling software, including Swiss model [58], pGen THREADER from Psi-Pred server [56], Phyre 2 [60], and Modeller [57], were deployed to generate the 3D structure of 5HT1A, as shown in Figure S1. LG and MaxSub score depend upon the target structure length; good quality long length structure possesses high LG score, whereas short length good quality structures have high MaxSub score. LG score and MaxSub score for all the generated models were found to be satisfactory; however, the MaxSub score for the Psi-Pred generated model was <0.1, indicating an incorrect model. Z-Score measured by ProSA-web service for the generated models lies within the range of scores observed for similar size native proteins [62]. ERRAT assess non-bonded interactions formed between different atom types to produce a score for incorrect regions. No incorrect region score was obtained for the model generated by MODELLER for 5HT1A. All the generated models were assessed through Ramachandran Plot using PROCHECK tool [65], which demonstrates that the SwissModel generated the best model with high scores in ‘Favoured’, ‘Additionally Allowed’, and ‘Generously Allowed’ regions as shown in Figures S2–S7. By interpreting the results based on ProQ [61], ProSA [62], ERRAT [63], and PROCHECK analysis, it was deduced that the structures developed by the SwissModel tool were better than the rest of the generated models (Figures S8 and S9) and can be used for further analysis, as shown in Table 6 and Figure 4.

### 3.4. Comparative Analysis of 5HT1A Structures

**Figure 5:**
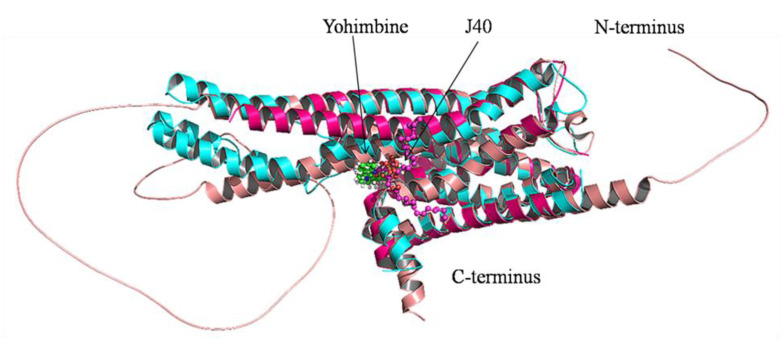
Cartoon representation of superimposed structures of 5HT1A. Homology model structure is shown in cyan color, which starts from Val31 and terminates at Lys418, yohimbine in green colored ball and sticks is shown bounded in the binding pocket. RCSB structure is shown in magenta color, which starts from Tyr35 and terminates at Ile415 with two long stretches of gaps, and J40 in magenta colored ball and sticks is shown bounded in the binding pocket. AlphaFold structure is shown in pink color, which starts from Met1 to Gln422 with a big loop.

Comparative analysis was conducted on the three-dimensional structure of 5HT1A retrieved through RCSB (PDB_ID:7E2Y_R) [76], through AlphaFold structure prediction, and homology modeled structure. The 7E2Y_R structure starts from Tyr35 and terminates at Ile415. It includes two stretches of gap; between Gly174 and Asp185, and between Lys228 and Ly324. The modeled structure by AlphaFold starts from Met1 to Gln422, with a loop of 77 residues from Gly243 to Ser320. However, our homology model structure starts from Val31 and terminates at Lys418. It is composed of alpha helices connected with loops from Thr229 to Arg323. Additionally, the predicted binding pocket of the modeled structure matches with the binding pocket of 7E2Y_R in which the ligands yohimbine and J40 bind, respectively, as shown in Figure 5.

### 3.5. Docking and Interaction Studies

**Figure 6:**
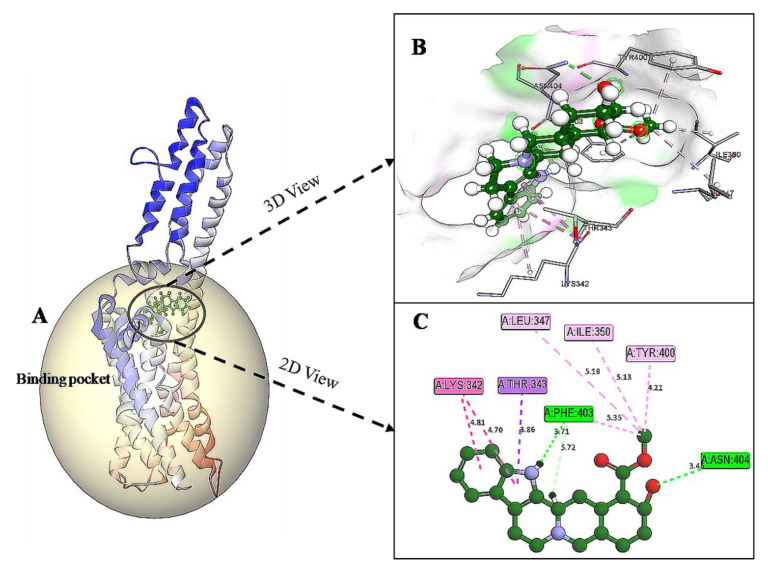
(**A**) Homology modeled structure of 5HT1A docked with yohimbine shown in ball and stick representation in green color within the binding site sphere of pink color. (**B**) Close intra-molecular interactions formed with the best ligand conformation. (**C**) 2D view of close intra-molecular interactions formed within the binding pocket residues and yohimbine as shown in green color ball and stick presentation, hydrogen bonds are shown in green dashed line, pi-sigma bonds in purple dashed line, and pi-alkyl bonds in pink dashed lines. The diagrams are prepared in Discovery Studio Client V 20.1.

The docking results of yohimbine and the TP model suggested the best ligand orientation of 46.65 GOLD Fitness Score fits into the predicted binding pocket. Ten confirmations of yohimbine were obtained, and the best conformation with the highest GOLD Fitness Score was selected for intra-molecular interaction studies. Close intra-molecular interactions formed by yohimbine within the binding pocket of 5HT1A model structure include hydrogen bond formation with Phe403, and Asn404, Pi-sigma bond with Thr343, amide Pi-stacking with Lys342, and alkyl interactions with Leu347, Ile350, and Tyr400, as shown in Figure 6.

### 3.6. In Silico Mutation Studies

**Figure 7:**
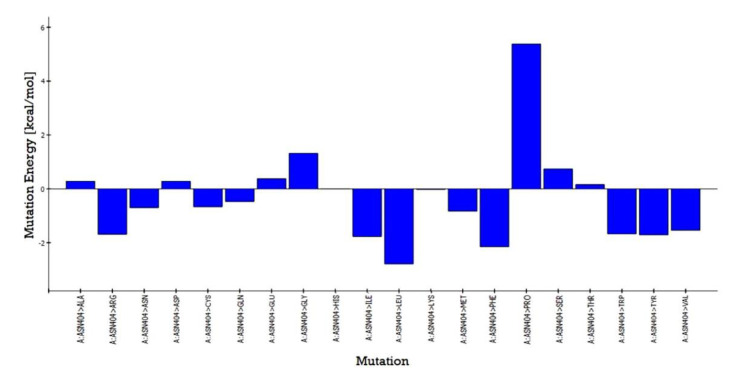
Asn404 mutation energy graph showing the lowest mutation energy when mutated to Leu to increase the stability of the model.

**Figure 8:**
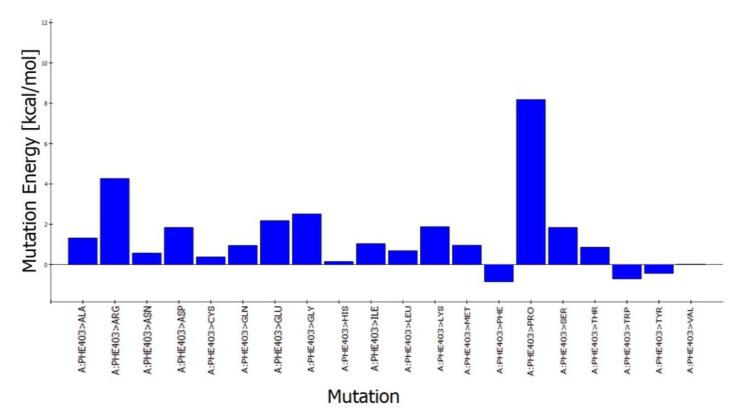
Phe403 mutation energy graph showing the lowest mutation energy when mutated to Phe to increase the stability of the model.

**Figure 9:**
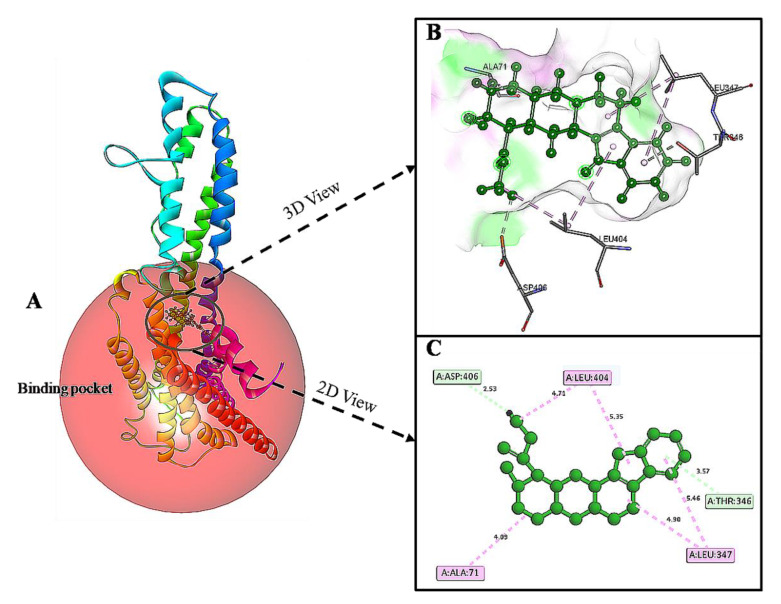
(**A**) Asn404Leu mutant model of 5HT1A docked with yohimbine shown in ball and stick representation in green color within the binding site sphere of pink color. (**B**) Close intra-molecular interactions formed with the best ligand conformation. (**C**) 2D view of close intra-molecular interactions formed within the binding pocket residues and yohimbine as shown in green color ball and stick presentation, Pi-donor hydrogen bonds and carbon-hydrogen bonds are shown in light green dashed line and pi-alkyl interactions in pink dashed lines. The diagrams are prepared in Discovery Studio Client v 20.1.

The predicted active-site residues of 5HT1A forming H-hydrogen bond with yohimbine were selected for the site-directed mutational study to analyze the effect of mutation on the stability of the generated protein structure. Phe403 and Asn404 forming hydrogen bonds with yohimbine were mutated to the residue contributing to the stability of the protein structure by using the ‘Build Mutant’ protocol of the DS. Asn404 when mutated to Arg, Cys, Gln, Ile, Leu, Met, Phe, Trp, Tyr, and Val was shown to increase the protein stability, as shown in Table 7. However, maximum stability was achieved when Asn404 was mutated to Leu with mutation energy of −2.79, as shown in Figure 7. While, when Phe303 was mutated to Phe, Trp, and Tyr it stabilized the structure of the TP. The lowest mutation energy change of −0.85 was observed when Phe403 was mutated to Phe, as shown in Figure 8. Therefore, the mutant model was prepared by considering Asn404Leu mutation. The mutant model was generated using the “Build and Edit Protein” module in DS to perform docking analysis within the predicted binding site using GOLD Suite to explore the bonds formed within the binding pocket residues. The generated model was energy minimized and CHARMm forcefield was applied to it. The ligand’s best binding conformation was estimated with a GOLD Fitness Score of 50.37, higher than the score observed for yohimbine binding with the WT model of 5HT1A. However, intra-molecular interactions formed within the binding pocket do not reveal hydrogen bond formation with the mutant residue at Leu404, which may be thermodynamically unfavorable for protein structure [77]. Mutant residue Leu404 is forming a hydrophobic interaction with yohimbine. Weak hydrogen bonds, carbon-hydrogen bonds and pi-donor hydrogen bonds are formed with Asp406 and Thr346, while hydrophobic interactions including alkyl and pi alkyl bonds are formed with Leu347, Leu404, and Ala71, as shown in Figure 9. Asn404Leu mutation increased the stabilization center of the TP as perceived by the GOLD fitness score, however, further increase in the stability of the mutant complex was assessed by MD simulation studies.

### 3.7. Molecular Dynamic Studies

MD simulations were performed to demonstrate the inhibition of the 5HT1A receptor and mutant 5HT1A by yohimbine. The analysis was comprehended by retrieving the trajectories root mean square deviation (RMSD), of root mean square fluctuation (RMSF), Radius of gyration (Rg), and solvent accessible surface area (SASA). The RMSD values of the Cα atoms in 5HT1A were plotted against the initial model across a 60 ns time scale and showed a sudden increase up to 1 ns, followed by a gradual increase up to 15 ns, and reached to a moderate equilibrium up to 60 ns that roughly falls within the range of 0.5–0.65 nm.

**Figure 10:**
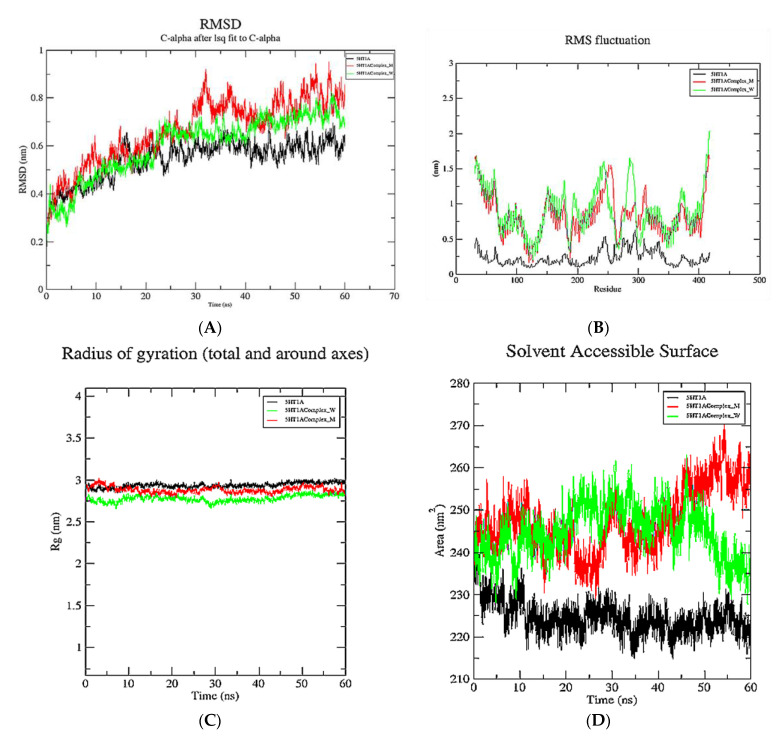
Graphical representation of 60 ns MD simulation analysis of 5HT1A modeled structure (in black color), wild type 5HT1A bound with yohimbine (in green color), and mutant 5HT1A bound with yohimbine (in red color). (**A**) RMSD values of Cα atoms in protein, (**B**) RMSF values of the backbone atoms, (**C**) radius of gyration the backbone atoms, and (**D**) SASA of the protein and complexes.

Root mean square deviation (RMSD) is measured to estimate the stability of the structure. Low RMSD refers to minimal conformational change and stability of the system during the simulation. To further understand the influence of the yohimbine on structural stability and integrity, the RMSD values of each yohimbine complex with wild type and mutant 5HT1A were also plotted. The RMSD values of the Cα atoms in complex of wild type 5HT1A and yohimbine show a rapid increase up to 1 ns, then increasing gradually up to 20 ns, followed by a moderate equilibrium up to 60 ns that roughly falls within the range of 0.6–0.8 nm. However, the RMSD values of the Cα atoms in complex of mutant type 5HT1A and yohimbine show a moderate increase up to 30 ns, followed by a state of moderate equilibrium up to 60 ns that roughly lies between 0.7 and 0.9 nm, as shown in Figure 10A.

The RMSF profile of the 5HT1A protein and yohimbine bound to wild type and mutant 5HT1A was analyzed, and the results revealed amino acid fluctuations in the binding and non-binding regions. The RMSF profile demonstrates fluctuations in the range of 0.25–0.5 nm at the 5HT1A binding site. Fluctuations in the wild type and mutant complexes were within the range of 0.5–1.5 nm; however, no significant fluctuations were observed at the yohimbine binding site in these complexes. Our study revealed an increase in overall fluctuations in the protein structure when bound with the ligand. The significant fluctuation between wild type and the mutant complex was observed in the non-binding region from 270–300 residues, which is not considered for the present study as the present study focuses on the binding regions in the protein, as shown in Figure 10B.

The radius of gyration (Rg) of the 5HT1A complex, as well as the wild type and mutant complexes with yohimbine, was also determined. The radius of gyration illustrates the compactness of the protein with protein folding and unfolding by applying thermodynamic principles. Rg values of all the systems lie within the range of 2.75–30 nm; however, Rg values of 5HT1A maintain equilibrium at 2.90 nm. Rg values of wild type 5HT1A complex with yohimbine started at 2.75 nm and increased up to 2.8 nm from 10 to 20 ns. A slight dip in Rg values was observed at 30 ns, which was resumed to 2.8 nm till 60 ns. Whereas, the Rg values of mutant 5HT1A complex with yohimbine began at 2.9 nm that increased up to 3 nm till 5 ns followed by a gradual decrease up to 2.8 nm to maintain equilibrium from 15 to 25 ns, a slight increase was observed at 30 ns which decreased and falls close to the Rg values of wild type 5HT1A complex with yohimbine. The analysis suggests that Rg values are higher for non-bound protein when compared to the yohimbine bound structures. Since the Rg values of the wild type and mutant complex structures with yohimbine do not significantly deviate from the non-bound structure, this suggests that there is a mild alteration in the microenvironment of the 5HT1A structure. This result implies a minimal change in the conformation of the protein structure when bound to yohimbine in wild type as well as in the mutant state, as shown in Figure 10C.

During the 60 ns simulation, the SASA of the 5HT1A and docked complexes of the yohimbine with wild type and mutant 5HT1A were examined and plotted. 5HT1A was exposed to the area of 215–235 nm^2^. The wild type 5HT1A complex with yohimbine was exposed to the surface area of 230–260 nm^2^; however, the mutant complex lies within the surface area of 230–270 nm^2^, as shown in Figure 10D.

### 3.8. Intra-Molecular Interactions during Simulation

**Figure 11:**
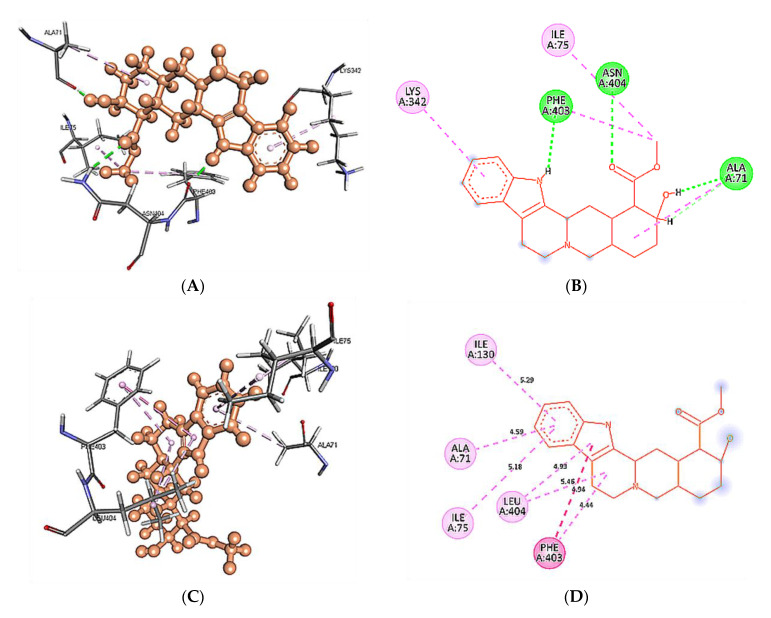
Intra-molecular interactions formed between docked complexes at 30 ns of MD simulation. (**A**,**B**) shows hydrogen bond in dashed line and hydrophobic bonds in pink dashed line, where yohimbine is presented in ball and stick while the interacting residues of wild type 5HT1A are shown in sticks. The figures are prepared in Discovery Studio Client V 20.1, in 3D and 2D formats, respectively. (**C**,**D**) shows interaction between mutant 5HT1A and yohimbine in 3D and 2D representations.

Intra-molecular interactions formed within docked complexes of wild type and mutant 5HT1A protein with yohimbine were analyzed during 60 ns simulation. It is apparent from interaction analysis that Phe403, Asn404, and Ala71 are forming hydrogen bonds with yohimbine at 30 ns. TheHD21 atom of Asn404 forms a conventional H-bond bond with O3 atom of yohimbine by donating an electron pair with a bond length of 2.60 Å. O atom of Phe403 forms a conventional H-bond of length by accepting an electron pair from H18 atom of yohimbine with a bond length of 1.99 Å. O atom of Ala71 also accepts an electron pair from H19 atom of yohimbine at the distance of 1.85 Å to form a conventional H-bond as shown in Figure 11A,B. Whereas, the mutant model complex with yohimbine does not form any H-bond at 30 ns while the mutant residueLeu404 was apparently observed to form hydrophobic interactions, as shown in Figure 11.

**Figure 12:**
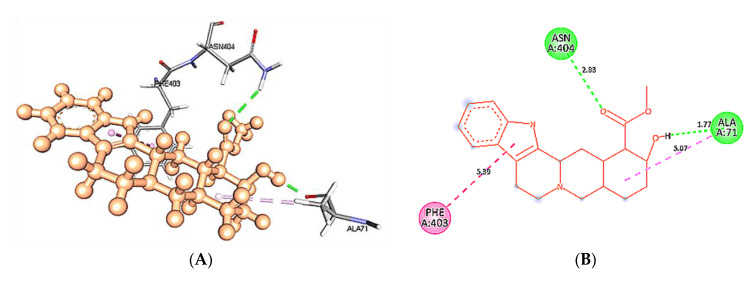

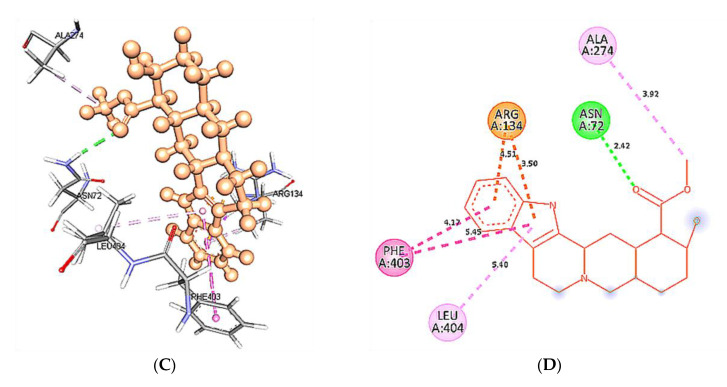
Intra-molecular interactions formed between docked complexes at 40 ns of MD simulation. (**A**,**B**) shows hydrogen bond in dashed line and hydrophobic bonds in pink dashed line, where yohimbine is presented in ball and stick while the interacting residues of wild type 5HT1A are shown in sticks. The figures are prepared in Discovery Studio Client V 20.1, in 3D and 2D formats, respectively. (**C**,**D**) shows interaction between mutant 5HT1A and yohimbine in 3D and 2D representations.

At 40 ns, Asn404 and Ala71 of wild type 5HT1A structure form H-bonds with yohimbine. HD21 atom of Asn404 forms conventional H-bond with yohimbine O3 by donating an electron pair at the distance of 2.82 Å. However, the HD22 atom of Asn72 forms a conventional H-bond with the O3 atom of yohimbine by donating an electron pair at a distance of 2.4 Å. Leu404 was apparently observed to form hydrophobic interaction, as shown in Figure 12.

**Figure 13:**
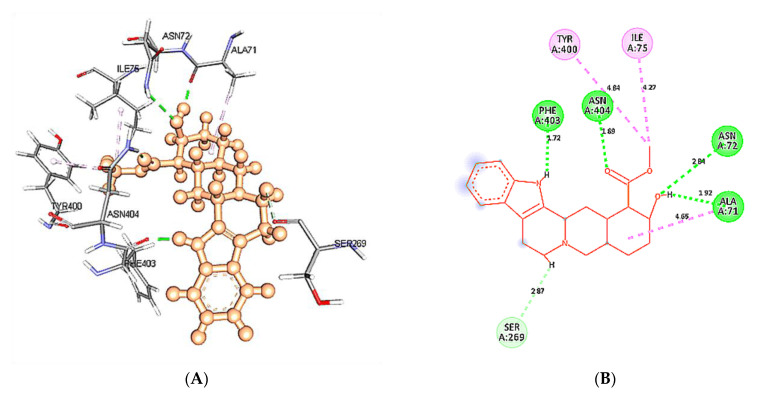

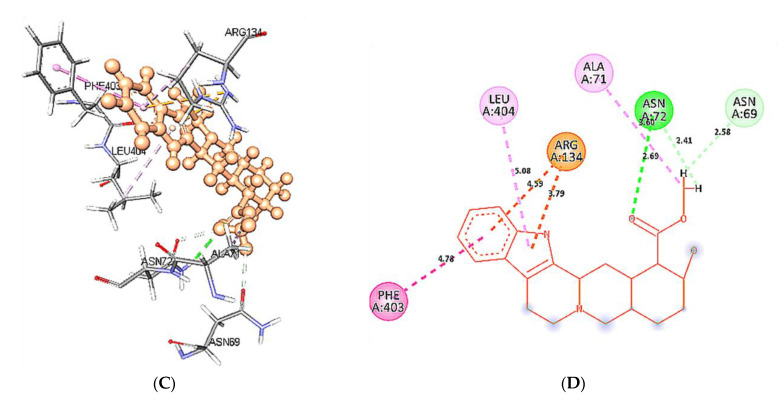
Intra-molecular interactions formed between docked complexes at 50 ns of MD simulation. (**A**,**B**) shows hydrogen bond in dashed line and hydrophobic bonds in pink dashed line, where yohimbine is presented in ball and stick while the interacting residues of wild type 5HT1A are shown in sticks. The figures are prepared in Discovery Studio Client V 20.1, in 3D and 2D formats, respectively. (**C**,**D**) shows interaction between mutant 5HT1A and yohimbine in 3D and 2D representations.

At 50 ns, Asn72, Asn404, Phe403, and Ala71 wild type 5HT1A structures are forming H-bonds with yohimbine. HD21 of Asn72 forms a conventional H-bond by donating an electron pair to the O3 atom of yohimbine at a distance of 2.8 Å. HD21 of Asn404 forms conventional H-bond at a distance of 1.89 Å with O3 of yohimbine by donating an electron pair. However, the O atom of Phe403 and Ala71 forms H-bond with H18 and H19 atom of yohimbine by accepting an electron pair at distances of 1.71 and 1.91 Å, respectively. Whereas, mutant 5HT1A forms only one conventional H-bond with yohimbine between HD22 atom of Asn72 and O3 atom of yohimbine by donating an electron pair at a distance of 2.6 Å. In addition, Leu404 is forming hydrophobic interactions with yohimbine, as shown in Figure 13.

**Figure 14:**
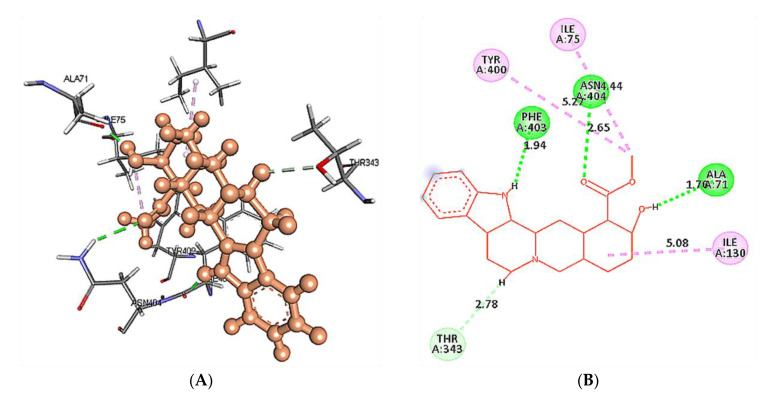

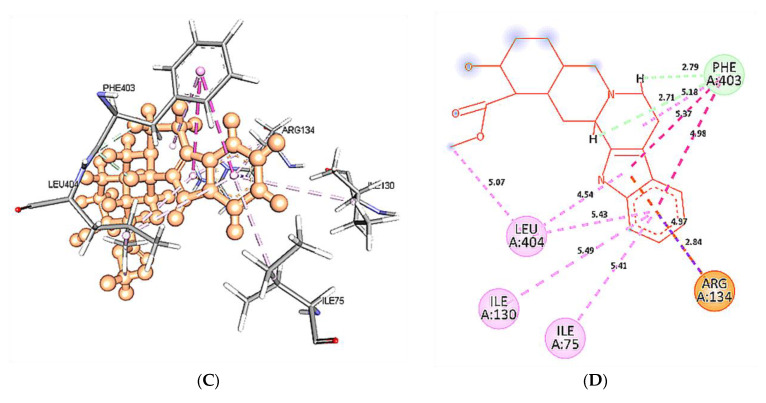
Intra-molecular interactions formed between docked complexes at 60 ns of MD simulation. (**A**,**B**) shows hydrophobic bonds in pink dashed line, where yohimbine is presented in ball and stick while the interacting residues of wild type 5HT1A are shown in sticks. The figures are prepared in Discovery Studio Client V 20.1, in 3D and 2D formats, respectively. (**C**,**D**) shows interaction between mutant 5HT1A and yohimbine in 3D and 2D representations.

At 60 ns, binding site residues form three conventional H-bonds in wild type 5HT1A structure and yohimbine docked complex. HD21 atom of Asn404 forms conventional H-bond by donating an electron pair to O3 atom of yohimbine. O atom of Phe403 and Ala71 forms conventional H-bond by accepting an electron pair from H18 and H19 from yohimbine at distances of 1.90 and 1.70 Å. Whereas, no H-bond formation is observed in mutant 5HT1A and yohimbine docked complex at 60 ns. The mutant residue Leu404 is involved in hydrophobic interactions, as shown in Figure 14. Hence, the present study demonstrates that yohimbine is able to form H-bonds in the binding pocket of wild type 5HT1A.

## 4. Conclusions

The present study was carried out to identify the role of yohimbine as a natural drug against the serotonergic receptor 5HT1A to treat MDD effectively. The best model structure of 5HT1A was generated using the Swiss Model. Yohimbine was found to possess drug-likeness and considerable good ADMET properties. A mutant model was constructed to increase the stability of the protein. The molecular docking results for wild type and mutant model revealed good binding scores. Wild-type model formed H-bonds with yohimbine, while the Asn404Leu mutant model formed hydrophobic interactions. Conformational changes and intra-molecular interactions were illustrated MD simulation studies. As a result of the mutation, there is no change in the conformation of mutants from their wild type structure, which is desirable for them to be biologically active. Our study provides an insight into yohimbine as a potential drug candidate for MDD. The presented in silico study needs to be further validated in the wet lab before clinical trials.

## Figures and Tables

**Table 1:** Computational parameters of pharmacokinetics (ADME) of yohimbine.

Property	Predicted Value
**Physicochemical properties**	
LogS (Solubility)	50.647 μg/mL
LogD_7.4_ (Distribution Coefficient D)	1.066
LogP (Distribution Coefficient P)	2.165
**Absorption**	
Papp (Caco-2 Permeability)	−4.783 cm/s
Pgp-inhibitor	+
Pgp-substrate	+
HIA (Human Intestinal Absorption)	+ (0)
F (20% Bioavailability)	+
F (30% Bioavailability)	+
**Distribution**	
PPB (Plasma Protein Binding	76.312 %
VD (Volume Distribution)	1.219 L/kg
BBB (Blood–Brain Barrier)	+++ (2)
**Metabolism**	
P450 CYP1A2 inhibitor	−−−
P450 CYP1A2 Substrate	−
P450 CYP3A4 inhibitor	−−−
P450 CYP3A4 substrate	++
P450 CYP2C9 inhibitor	−−−
P450 CYP2C9 substrate	−−−
P450 CYP2C19 inhibitor	−−−
P450 CYP2C19 substrate	−
P450 CYP2D6 inhibitor	++
P450 CYP2D6 substrate	++
**Elimination**	
T_1/2_ (Half Life Time)	1.615 h
CL (Clearance Rate)	2.321 mL/min/kg

Explanation: ‘+’ Low; ‘++’ Moderate; ‘+++’ High; ‘−’ Low and ‘−−−’ High.

**Table 2:** Physicochemical properties of yohimbine.

Compounds	Rotatable Bonds	MW (<500)	ALog P (≤5)	H-Bond Donor (≤5)	H-Bond Acceptor (≤10)	Rule of 5 Violations
Yohimbine	2	354.443	2.82	2	5	0

Abbreviations: MW, molecular weight; LogP, octanol/water partition coefficient.

**Table 3:** Carcinogen and toxic evaluation of yohimbine.

Parameters	Unit	Yohimbine
Rat inhalation LC_50_	mg/m^3^/h	3590.05
Rate of oral LD_50_	g/kg body weight	0.311154
Rat chronic LOEAL	g/kg body weight	0.00473724
Daphnia EC_50_	mg/mL	4.13671
Fathead minnow LC_50_	g/L	0.0106882
hERG (hERG Blockers)		+
H-HT (Human Hepatotoxicity)		−
DILI (Drug Induced Liver Injury)		−
**Carcinogenic potency TD_50_**		
Rat	mg/kg body weight/day	0.00302322
Mouse	mg/kg body weight/day	27.5687
Rat maximum tolerated dose	g/kg body weight	0.0664294
Developmental toxicity potential		Toxic
Aerobic biodegradability		Non-degradable
Ames mutagenicity		Non-mutagen
Skin irritancy		Mild
Ocular irritancy		Severe

**Table 4:** Estimation of physico-chemical parameters of 5HT1A.

Target Protein	UniProt ID	Residues	Molecular Weight	Theoretical pI	Instability Index	Aliphatic Index	Grand Average of Hydropathicity (GRAVY)
5HT1A	P08908	422	46106.88	9.13	36.52	100.81	0.195

**Table 5:** Prediction of post-translational modification sites in5HT1A.

Target Protein	Residue	Post-Translational Modification
5HT1A	T196	Phosphorylation
	S199	Phosphorylation
	Y205	Phosphorylation
	Y215	Phosphorylation
	T240	Phosphorylation
	K324	Acetylation
	K334	Ubiquitination
	T60	Phosphorylation

**Table 6:** Evaluation scores for the generated 3D structures of the target proteins.

Target Protein	Tool	ProQ		ProSA ZScore	ERRAT	PROCHECK (in %)		
		LGscore	MaxSub			F	A	G
5HT1A	Swiss Model	2.238	0.139	−5.29	88.740	94.5	4.4	1.0
	MODELLER	11.480	1.071	0.64	n.a.	96.4	3.09	0.47
	Psi-Pred	1.770	0.068	n.a.	71.498	70	19.1	10.8
	Phyre 2	2.035	0.129	−1.18	80.323	93.6	3.4	2.9

Abbreviations: F, favored; A, allowed; G, generously allowed region.

**Table 7:** ASN404 mutation energy and stability analyzed by Build Mutant tool.

Mutation	Mutation Energy	Effect of Mutation
A:ASN404>ALA	0.28	neutral
A:ASN404>ARG	−1.69	stabilizing
A:ASN404>ASN	−0.7	stabilizing
A:ASN404>ASP	0.28	neutral
A:ASN404>CYS	−0.67	stabilizing
A:ASN404>GLN	−0.47	neutral
A:ASN404>GLU	0.38	neutral
A:ASN404>GLY	1.32	destabilizing
A:ASN404>HIS	0	neutral
A:ASN404>ILE	−1.77	stabilizing
A:ASN404>LEU	−2.79	stabilizing
A:ASN404>LYS	−0.02	neutral
A:ASN404>MET	−0.83	stabilizing
A:ASN404>PHE	−2.15	stabilizing
A:ASN404>PRO	5.38	destabilizing
A:ASN404>SER	0.74	destabilizing
A:ASN404>THR	0.16	neutral
A:ASN404>TRP	−1.67	stabilizing
A:ASN404>TYR	−1.71	stabilizing
A:ASN404>VAL	−1.54	stabilizing

## Data Availability

Not applicable.

## Supplementary Materials

The following are available online at https://www.mdpi.com/article/10.3390/cimb43030127/s1.
