# Supplementary material for: Investigation of Antidepressant Properties of Yohimbine by Employing Structure-Based Computational Assessments
Source: Curr Issues Mol Biol. 2021 Oct 27;43(3):1805–27. doi: 10.3390/cimb43030127 (PMC8929124; doi:10.3390/cimb43030127)
Supplement: Supplementary file 1 [file cimb-43-00127-s001.zip › cimb-1415107-supplementary.pdf]

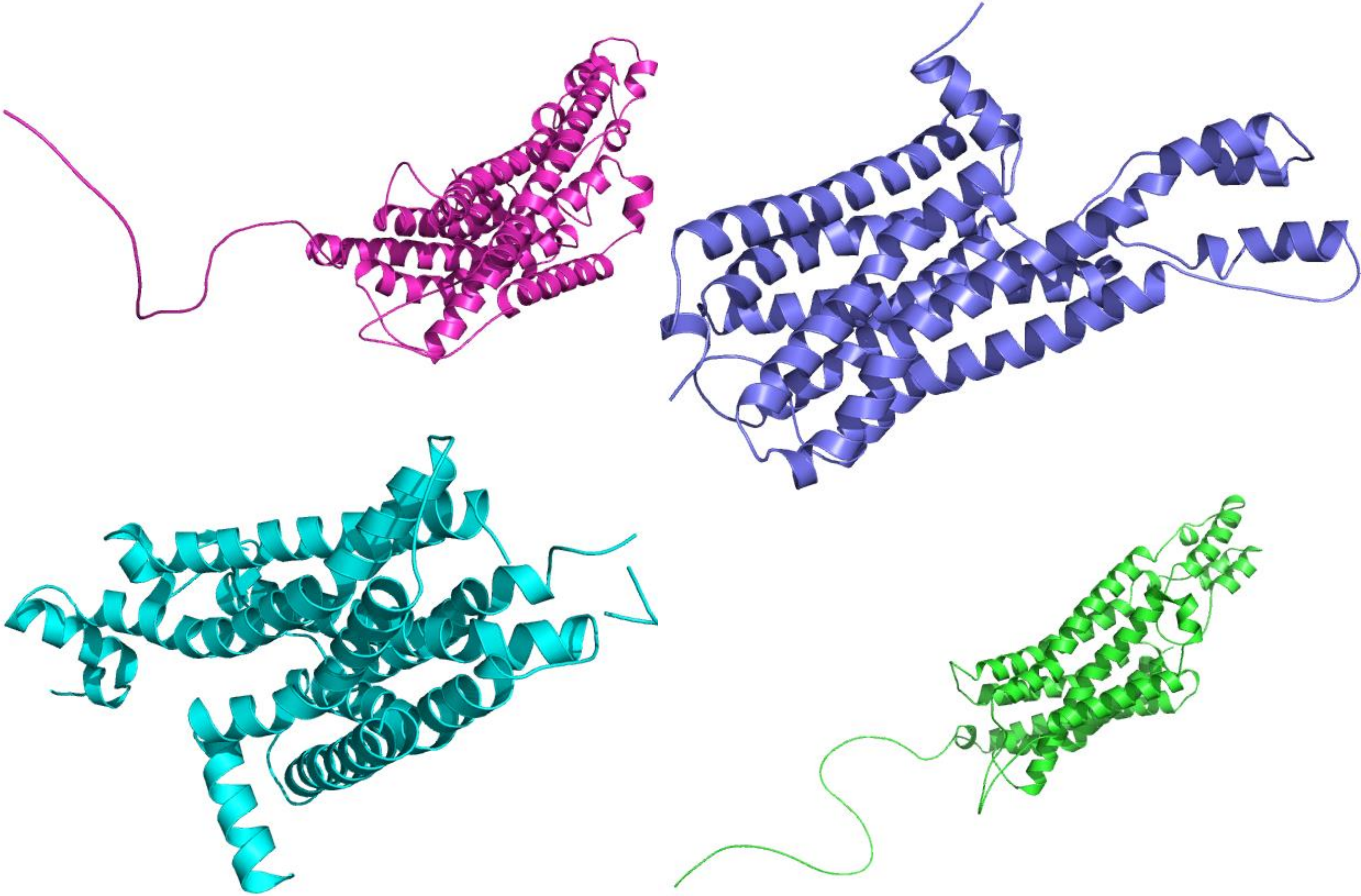

Figure S1: Generated models of 5HT1B using a) PsiPred as shown in magenta color, b) SwissModel as shown in purple color, c) Phyre2 as shown in cyan color, and d) Modeller as shown in green color.

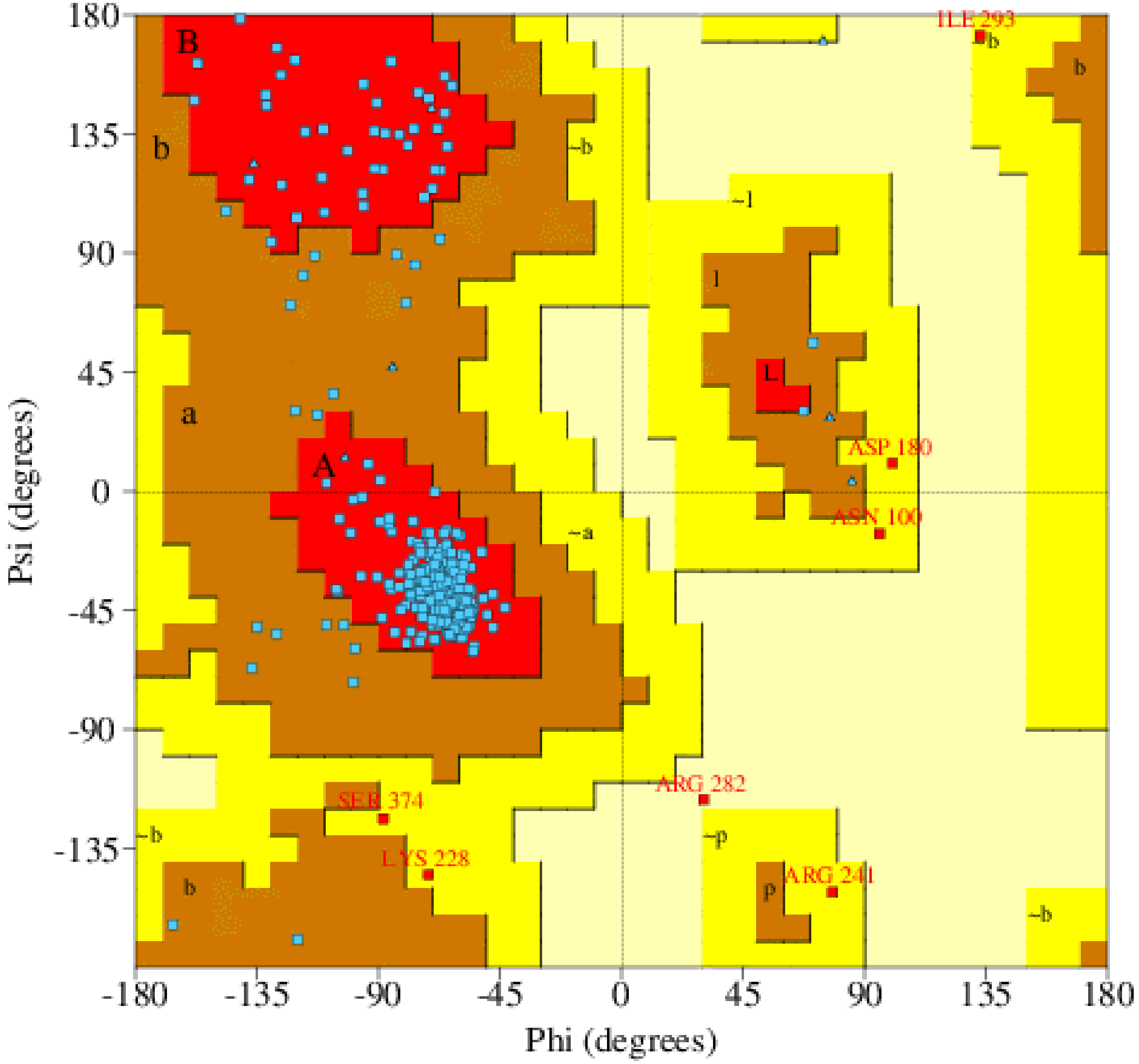

Figure S2 : Ramachandran Plot obtained for the Phyre2 generated model indicating 91.9% residues in most favored region, 6.0% residues in additionally allowed region, 1.8% in generously allowed region and only 0.3 % in not allowed region with satisfactory G-factor value of 0.16.

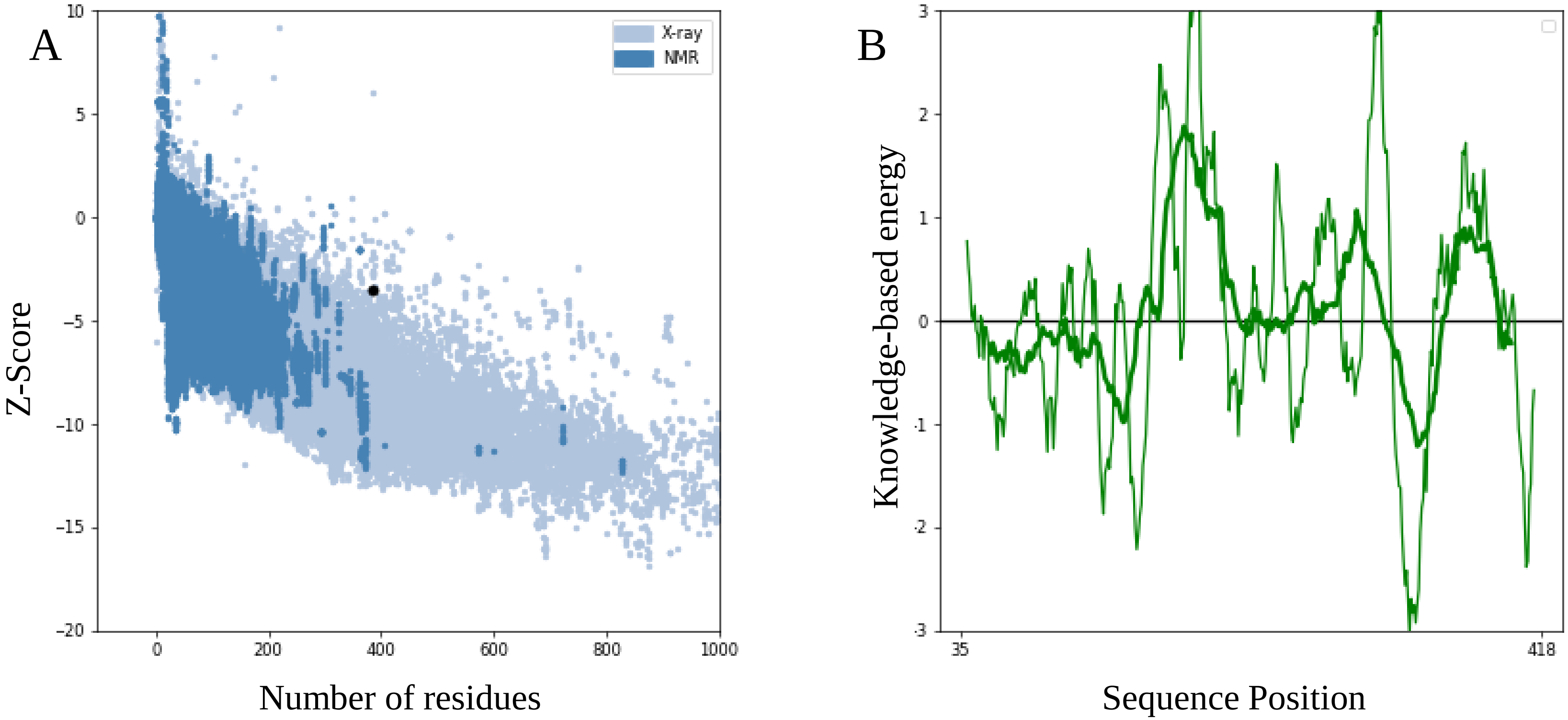

Figure S3: Investigation of 5HT1A model by Phyre2 using the ProSA-web service. A) Z-score of 5HT1A model generated by Phre2 is highlighted with black dot with a satisfactory score of -3.54 that falls close to the range scores observed for similar size native proteins. B) Energy plot of 5HT1A model. Residue energies are averaged over each 40-residue and plotted as a function of the central residue in the window size of 40 as shown in dark green color. Many parts of the structure falls in the positive range to represent erroneous segments in the generated model.

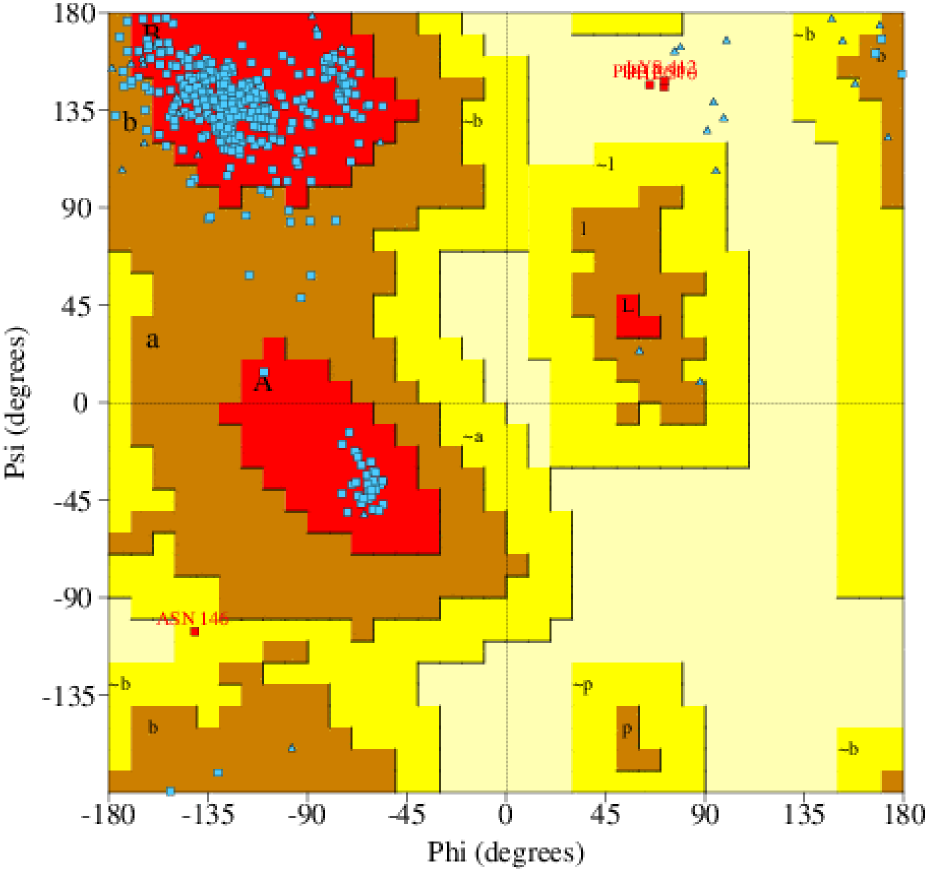

Figure S4: Ramachandran Plot obtained for the Modeller generated model indicating 90.2% residues in most favored region, 8.7% residues in additionally allowed region, 0.3% in generously allowed region and only 0.8 % in not allowed region with satisfactory G-factor value of -0.16.

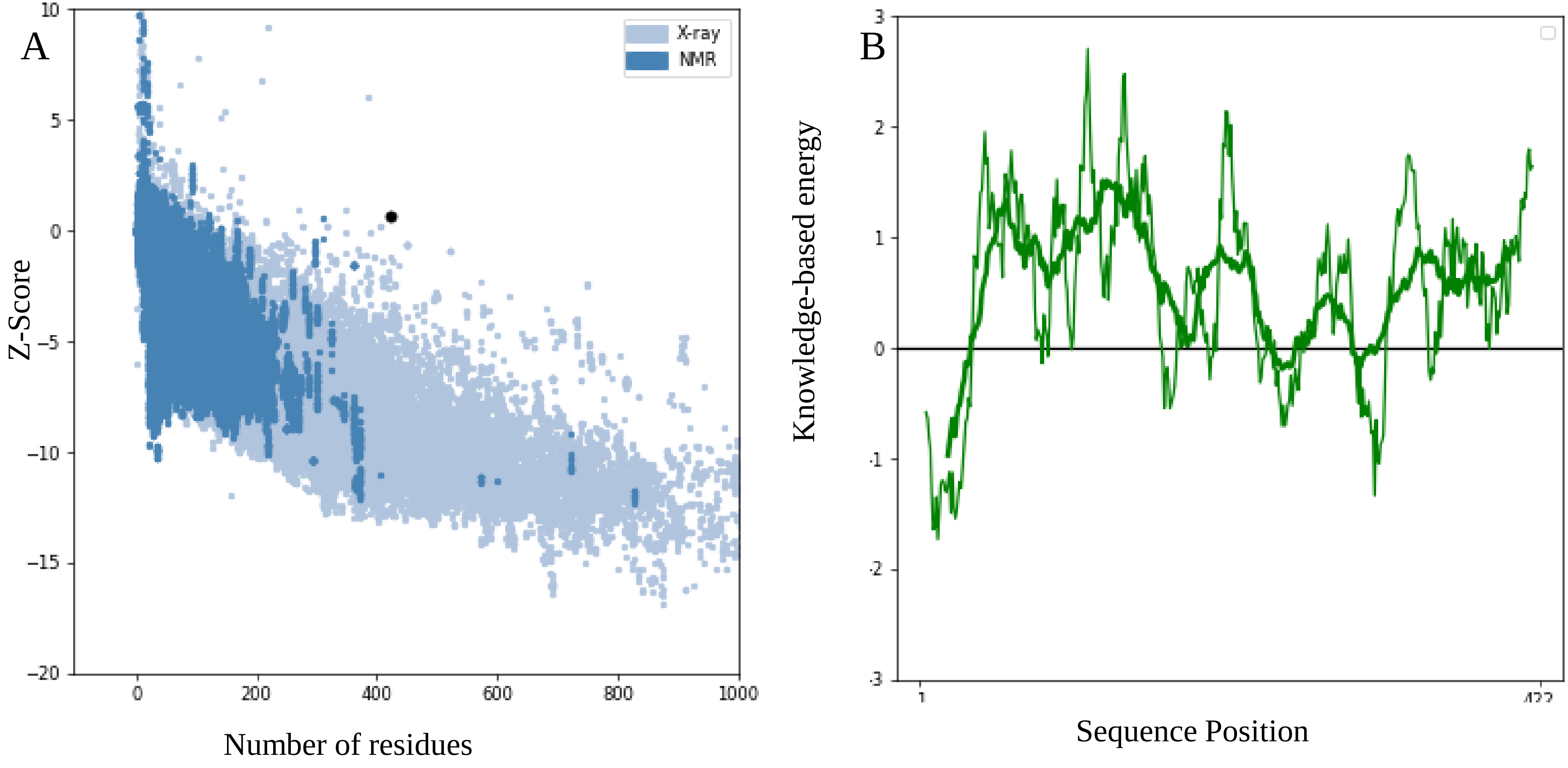

Figure S5: Investigation of 5HT1A model by Modeller using the ProSA-web service. A) Z-score of 5HT1A model generated by Modeller is highlighted with black dot of score 0.64 that falls away from the range scores observed for similar size native proteins. B) Energy plot of 5HT1A model. Residue energies are averaged over each 40-residue and plotted as a function of the central residue in the window size of 40 as shown in dark green color. Most of the structure falls in the positive range to represent erroneous segments in the generated model.

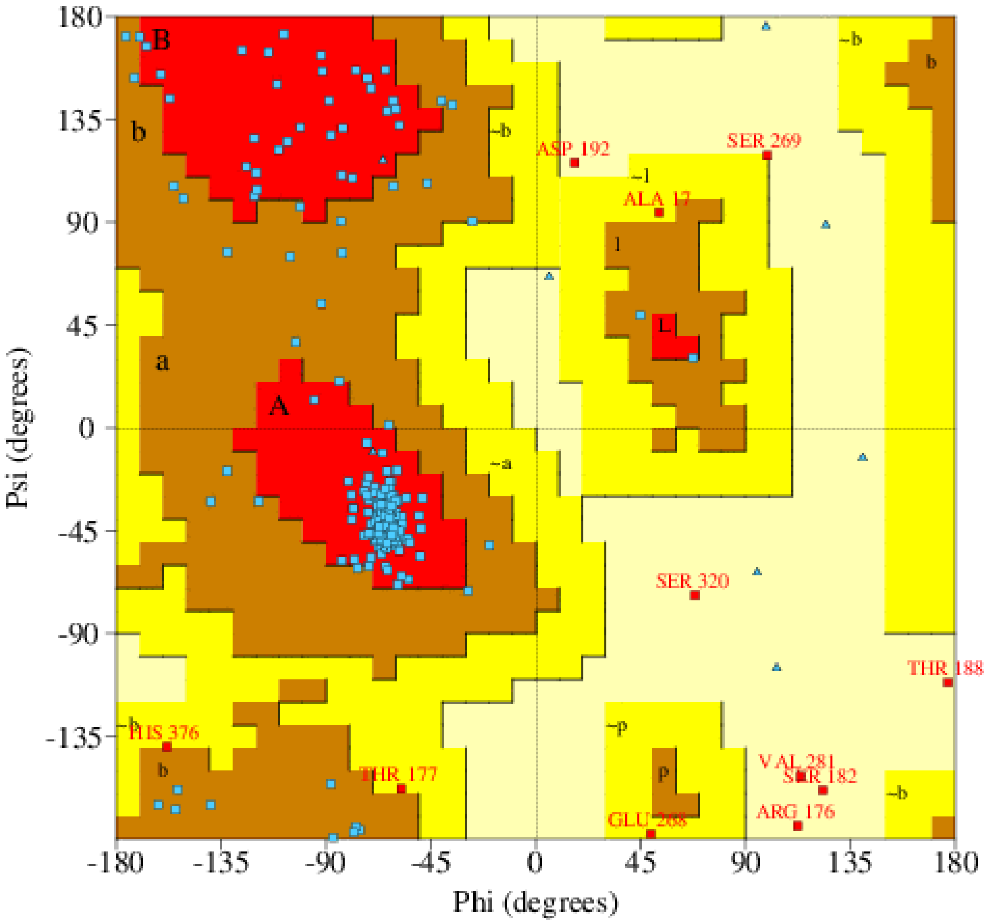

Figure S6: Ramachandran Plot obtained for the pGenTHREADER generated model indicating 88% residues in most favored region, 9.0% residues in additionally allowed region, 1.4% in generously allowed region and only 1.6 % in not allowed region with satisfactory G-factor value of -0.26.

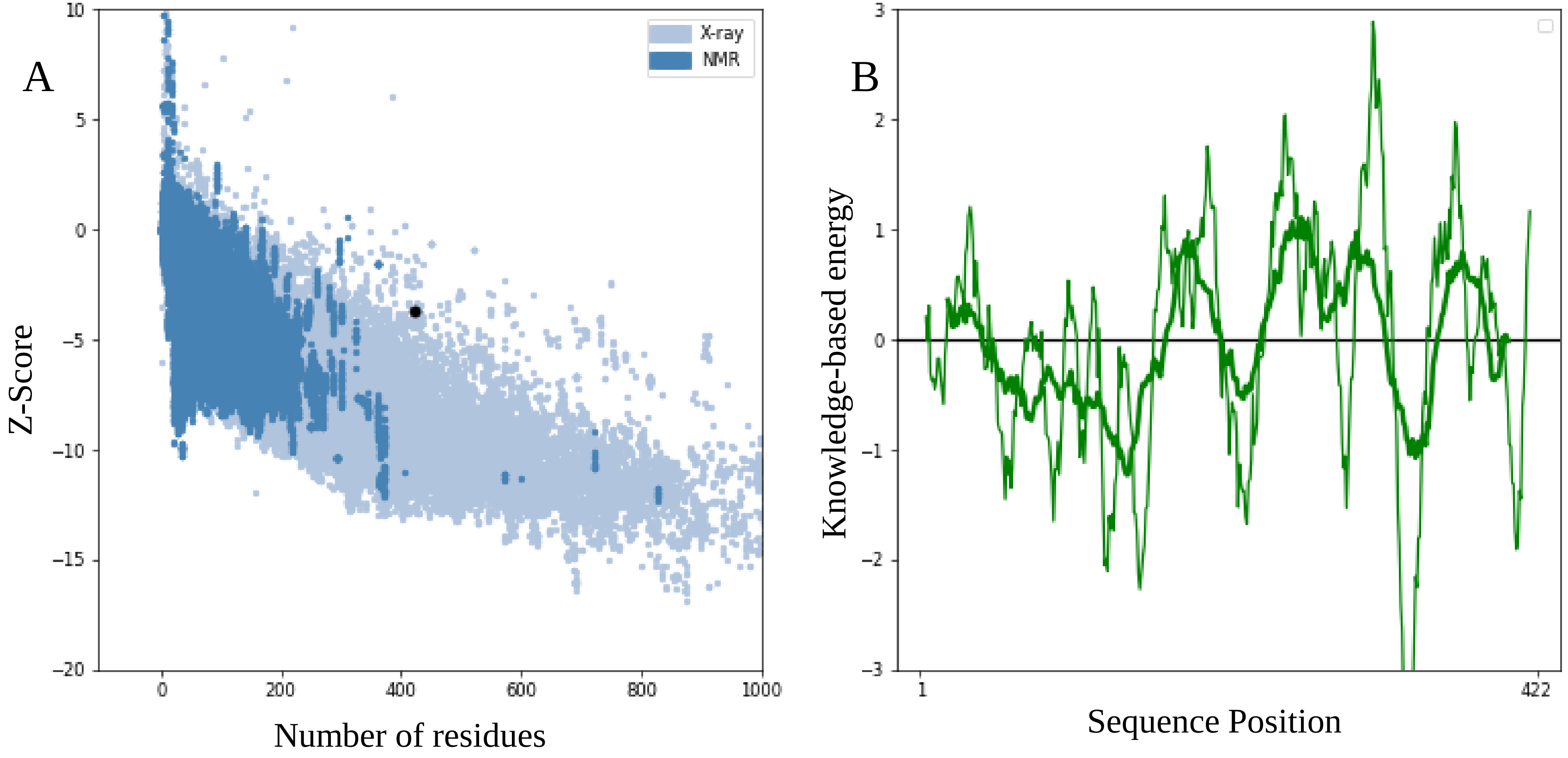

Figure S7: Investigation of 5HT1A model by pGenTHREADER using the ProSA-web service. A) Z-score of 5HT1A model generated by pGenTHREADER is highlighted with black dot of score -3.66 that falls in the proximity of the range scores observed for similar size native proteins. B) Energy plot of 5HT1A model. Residue energies are averaged over each 40-residue and plotted as a function of the central residue in the window size of 40 as shown in dark green color. Many regions of the structure falls in the positive range to represent erroneous segments in the generated model.

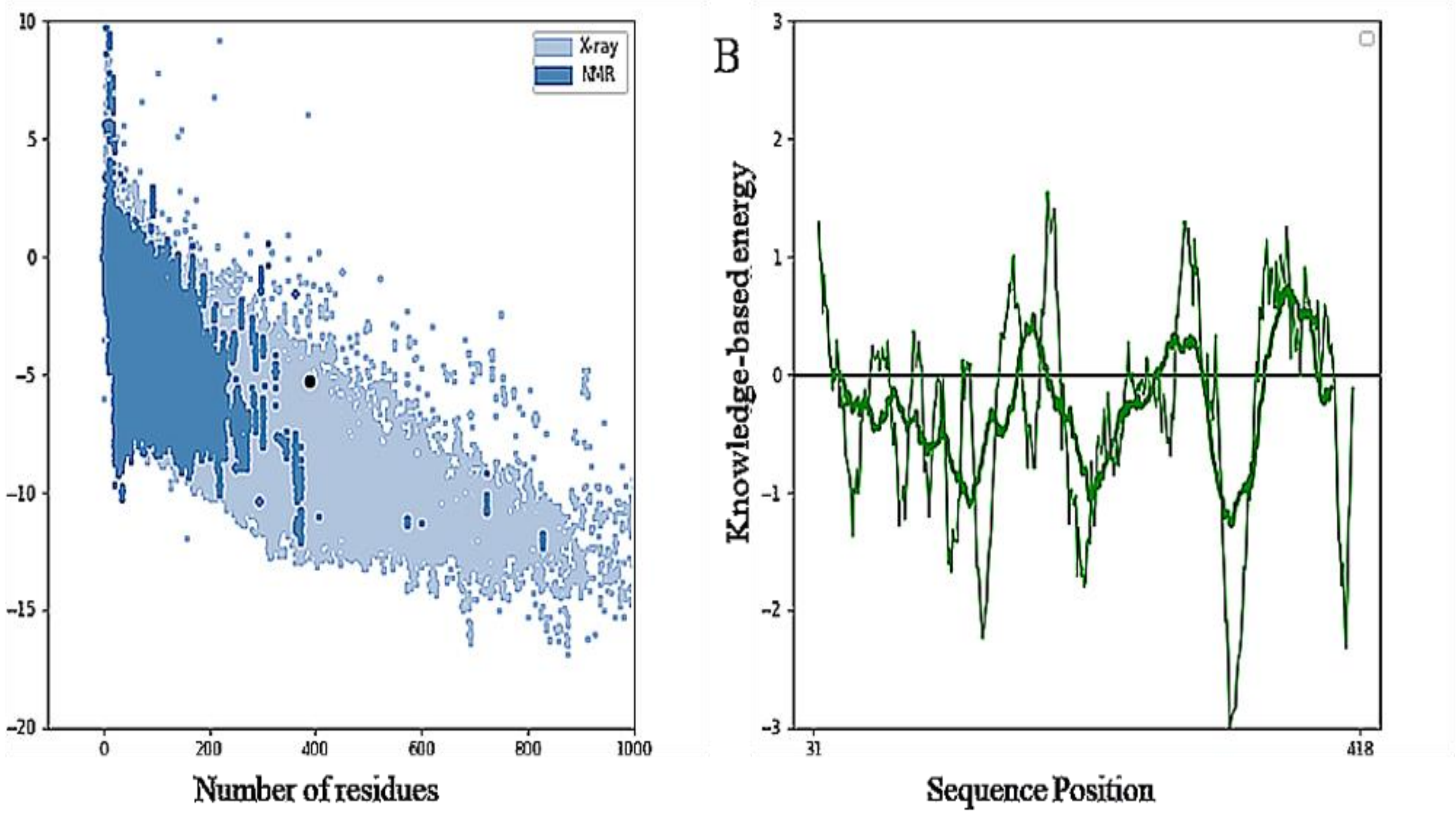

Figure S8: Investigation of 5HT1A model using the ProSA-web service. A) Z-score of 5HT1A model generated by SwissModelis highlighted with black dot with a satisfactory score of -5.29 that falls within the range scores observed for similar size native proteins. B) Energy plot of 5HT1A model. Residue energies are averaged over each 40-residue and plotted as a function of the central residue in the window size of 40 as shown in dark green color. Few parts of the structure lies in the positive range to represent erroneous segments in the generated model. However, major portion of the model falls in the negative range of good quality region.

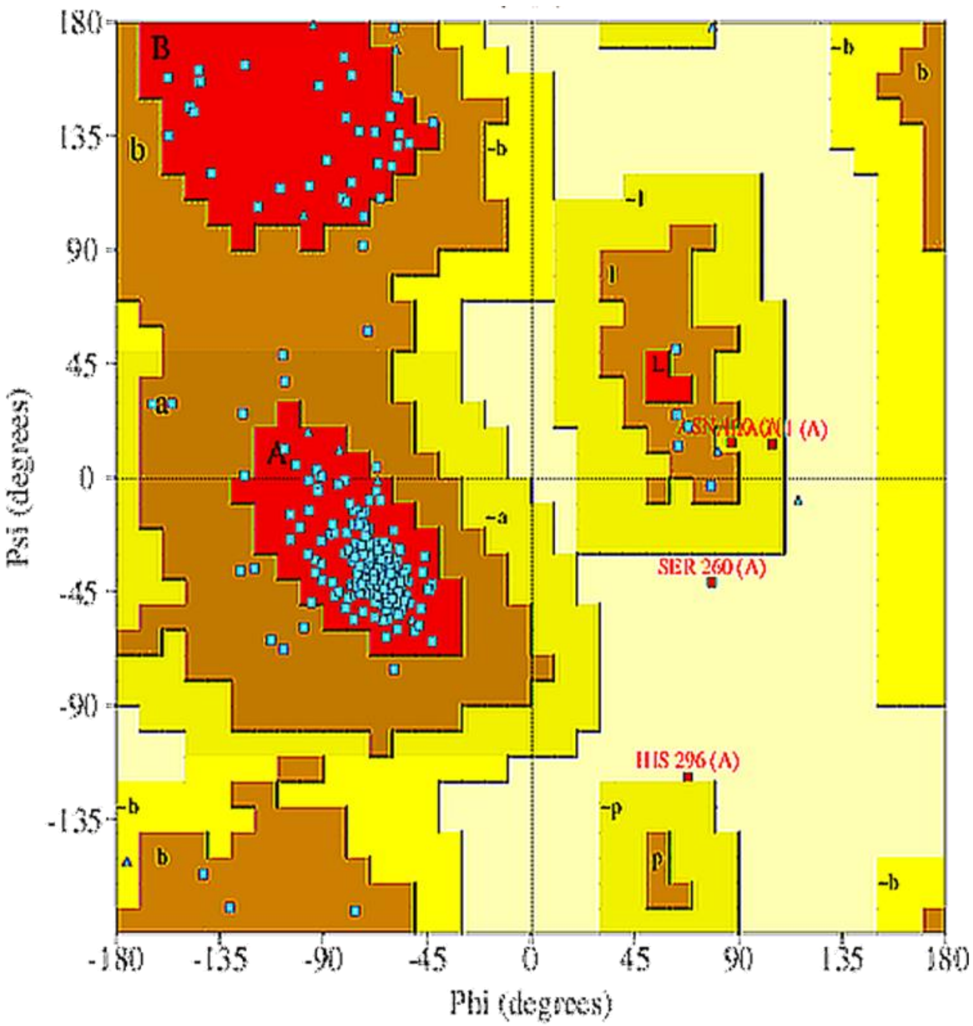

Figure S9: Ramachandran Plot obtained for the SwissModel generated model indicating 91.8% residues in most favored region, 7.0% residues in additionally allowed region, 0.6% in generously allowed region and only 0.6 % in not allowed region with satisfactory G-factor value of -0.14.
